# Supplementary material for: Dietary Behaviors and Incident COVID-19 in the UK Biobank
Source: Nutrients. 2021 Jun 20;13(6):2114. doi: 10.3390/nu13062114 (PMC8234071; doi:10.3390/nu13062114)
Supplement: Supplementary file 1 [file nutrients-13-02114-s001.zip › nutrients-1241695-supplementary.pdf]

Supplemental Table 1: Description of the dietary touchscreen questionnaire and possible responses

Computed Variables	Question Stem	Responses	Hints
Coffee consumption, cups/day	How many cups of coffee do you drink each DAY? (Include decaffeinated coffee)	Enter INTEGER OR<br>Less than one OR<br>Do not know OR<br>Prefer not to answer	Please provide an average considering your intake over the last year. If you are unsure, please provide an estimate or select Do not know.
Tea consumption, cups/day	How many cups of tea do you drink each DAY? (Include black and green tea)	Enter INTEGER OR<br>Less than one OR<br>Do not know OR<br>Prefer not to answer	Please provide an average considering your intake over the last year. If you are unsure, please provide an estimate or select Do not know.
Fruit (fresh/dried), servings/day	About how many pieces of FRESH fruit would you eat per DAY? (Count one apple, one banana, 10 grapes etc as one piece; put '0' if you do not eat any)<br>About how many pieces of DRIED fruit would you eat per DAY? (Count one prune, one dried apricot, 10 raisins as one piece; put '0' if you do not eat any)	Enter INTEGER OR<br>Less than one OR<br>Do not know OR<br>Prefer not to answer	Please provide an average considering your intake over the last year. If you are unsure, please provide an estimate or select Do not know
Vegetables (cooked/raw), servings/day	On average how many heaped tablespoons of COOKED vegetables would you eat per DAY? (Do not include potatoes; put '0' if you do not eat any)<br>On average how many heaped tablespoons of SALAD or RAW vegetables would you eat per DAY? (Include lettuce, tomato in sandwiches; put '0' if you do not eat any)	Enter INTEGER OR<br>Less than one OR<br>Do not know OR<br>Prefer not to answer	Please provide an average considering your intake over the last year. If you are unsure, please provide an estimate or select Do not know. If you have less than one tablespoon a day select Less than one.
Oily fish consumption, servings/day	How often do you eat oily fish? (e.g. sardines, salmon, mackerel, herring)	SELECT one:<br>Never<br>Less than once a week<br>Once a week<br>2-4 times a week<br>5-6 times a week<br>Once or more daily<br>Do not know<br>Prefer not to answer	Please provide an average considering your intake over the last year. If you are unsure, please provide an estimate or select Do not know. Oily fish include: Salmon, Anchovies, Trout, Swordfish, Mackerel, Bloater, Herring, Cacha, Sardines, Carp, Pilchards, Hilsa, Kipper, Jack fish, Eel, Katla,Whitebait, Orange roughy, Tuna (fresh only), Pangas, Sprats.
Processed meat, servings/day	How often do you eat processed meats (such as bacon, ham, sausages, meat pies, kebabs, burgers, chicken nuggets)?	SELECT one:<br>Never<br>Less than once a week<br>Once a week<br>2-4 times a week	Please provide an average considering your intake over the last year If you are unsure, please provide an estimate or select Do not know.

		5-6 times a week<br>Once or more daily<br>Do not know<br>Prefer not to answer	
Red meat, servings/day	How often do you eat beef? (Do not count processed meats)<br>How often do you eat lamb/mutton? (Do not count processed meats)<br>How often do you eat pork? (Do not count processed meats such as bacon or ham)	SELECT one:<br>Never<br>Less than once a week<br>Once a week<br>2-4 times a week<br>5-6 times a week<br>Once or more daily<br>Do not know<br>Prefer not to answer	Please provide an average considering your intake over the last year If you are unsure, please provide an estimate or select Do not know
Breastfed as a baby	Were you breastfed when you were a baby?	SELECT one:<br>Yes<br>No<br>Do not know<br>Prefer not to answer	

Source: https://biobank.ndph.ox.ac.uk/showcase/showcase/docs/TouchscreenQuestionsMainFinal.pdf

Supplemental Table 2. Characteristics of the analysis sample by age groups

Characteristics*	Age Group	
	**Age <55**	**Age ≥ 55**
**Number of persons**	**13,309**	**2,4679**
Age, yr, mean(sd)	47.64 (4.16)	62.60 (4.08))
Female	7,646 (57.45)	12,380 (50.16))
Townsend-deprivation index, mean(sd)	-0.82*(3.26)	-1.42 (3.01)
White/British	12,074 (90.72)	23,719 (96.11)
Household Income, £< 18,000	1,817 (13.65)	6,447 (26.12)
College or university degree	4,551 (34.19)	6,449 (26.13)
Currently employed	11,353 (85.30)	9,001 (36.47)
Homeowner	11,842 (88.98)	22,453 (90.98)
Number of co-habitants ≥ 4	5,176 (38.89)	1,645 (6.67)
Current smoker	1,926 (14.47)	2,279 (9.23)
BMI (kg/m^2), mean(sd)	27.70* (5.21)	28.05 (4.79)
Physical activity, minutes/day, mean(sd)	75.76 (100.10)	75.36 (96.10)
Breastfed as baby	6,998 (52.58)	14,053 (56.94)
Poor overall health rating	881 (6.62)	1,531 (6.20)
Using cholesterol medication	1,051 (7.90)	7,144 (28.95)
Using blood pressure medication	1,470 (11.05)	8,140 (32.98)
Diabetes	524 (3.94)	2,156 (8.74)
Heart diseases	363 (2.73)	2,640 (10.70)
Coffee consumption, cups/day, mean(sd)	2.04 (2.19)	2.01(1.98)
Tea consumption, cups/day, mean(sd)	3.28* (2.81)	3.51 (2.69)
Fruit (fresh/dried), servings/day, mean(sd)	2.68* (2.44)	3.24 (2.67)
Vegetables (cooked/raw), servings/day, mean(sd)	0.79* (0.56)	0.84 (0.54)
Oily fish consumption, servings/day, mean(sd)	0.14* (0.14)	0.17 (0.16)
Processed meat, servings/day, mean(sd)	0.22* (0.21)	0.21 (0.20)
Red meat, servings/day, mean(sd)	0.29* (0.21)	0.31 (0.21)
Pre-existing compromised pulmonary function	6,581 (49.45)	13,549 (54.90)
COVID-19 exposed rate**, mean(sd)	3.33 (1.49)	3.22 (1.49)
COVID_19 positive	3,439 (25.84)	3,043 (12.33)

*Data drawn from baseline (2006-10) except for pre-existing compromised pulmonary function, the COVID-19 exposed rate, and COVID_19 test results. Values are numbers (%) unless stated otherwise. All characteristic values are significantly different across age groups (P<0.05), except for physical activity and coffee consumption.

**Average monthly rates (per 1000) of positive cases per specific geo-populations between March and November 2021.

Supplemental Table 3. Characteristics of the analysis sample by racial groups

Characteristics*	White	Asian	Black	Mixed/Others
**Number of persons**	**35793**	**899**	**638**	**658**
Age, yr, mean(sd)	57.59 (8.17)	54.06 (8.46)	53.07 (8.16)	53.24 (8.19)
Female	18,830 (52.61)	413 (45.94)	393 (61.60)	390 (59.27)
Townsend-deprivation index, mean(sd)	-1.35 (3.03)	0.31 (3.23)	2.65 (3.44)	0.74 (3.73)
Household Income, £< 18,000	7,710 (21.54)	214 (23.80)	178 (27.90)	162 (24.62)
College or university degree	10,168 (28.41)	343 (38.15)	213 (33.39)	276 (41.95)
Currently employed	18,969 (53.00)	545 (60.62)	422 (66.14)	418 (63.53)
Homeowner	32,608 (91.10)	788 (87.65)	407 (63.79)	492 (74.77)
Number of co-habitants ≥ 4	5,986 (16.72)	462 (51.39)	180 (28.21)	193 (29.33)
Current smoker	3,922 (10.96)	102 (11.35)	77 (12.07)	104 (15.81)
BMI (kg/m^2), mean(sd)	27.90 (4.93)	27.24 (4.62)	29.80 (5.39)	28.34 (5.35)
Physical activity, minutes/day, mean(sd)	76.20 (97.57)	54.79 (79.21)	74.70 (114.39)	66.80 (96.07)
Poor overall health rating	2,203 (6.15)	95 (10.57)	52 (8.15)	62 (9.42)
Using cholesterol medication	7,678 (21.45)	249 (27.70)	139 (21.79)	129 (19.60)
Using blood pressure medication	8,928 (24.94)	272 (30.26)	241 (37.77)	169 (25.68)
Diabetes	2,324 (6.49)	181 (20.13)	94 (14.73)	81 (12.31)
Heart diseases	2,830 (7.91)	94 (10.46)	45 (7.05)	34 (5.17)
Breastfed as baby	19,388 (54.17)	713 (79.31)	526 (82.45)	424 (64.44)
Coffee consumption, cups/day, mean(sd)	2.07 (2.07)	0.86 (1.28)	1.05 (1.49)	1.64 (1.79)
Tea consumption, cups/day, mean(sd)	3.47 (2.76)	2.86 (1.95)	2.50 (2.12)	2.88 (2.57)
Fruit (fresh/dried), servings/day, mean(sd)	3.00 (2.51)	3.97 (3.96)	3.74 (4.05)	3.58 (3.10)
Vegetables (cooked/raw), servings/day, mean(sd)	0.82 (0.53)	1.04 (0.82)	0.88 (0.64)	0.97 (0.71)
Oily fish consumption, servings/day, mean(sd)	0.16 (0.15)	0.12 (0.14)	0.22 (0.21)	0.18 (0.17)
Processed meat, servings/day, mean(sd)	0.22 (0.20)	0.16 (0.20)	0.17 (0.20)	0.16 (0.19)
Red meat, servings/day, mean(sd)	0.30 (0.20)	0.23 (0.24)	0.35 (0.28)	0.32 (0.25)
Pre-existing compromised pulmonary function	18,970 (53.00)	519 (57.73)	309 (48.43)	332 (50.46)
COVID-19 exposed rate**	3.28 (1.48)	2.90 (1.47)	2.72 (1.55)	2.90 (1.59)
COVID_19 positive	6,011 (16.79)	223 (24.81)	123 (19.28)	125 (19.00)

*Data drawn from baseline (2006-10) except for pre-existing compromised pulmonary function, COVID-19 exposed rate, and COVID_19 test results. Values are numbers (%) unless stated otherwise. All characteristic values are significantly different across racial groups (P<0.001).
**Average monthly rates (per 1000) of positive cases per specific geo-populations between March and November 2021.

Supplemental Table 4: Age-race-sex adjusted OR (95% CI) of having positive COVID-19 test by nutritional factors

Nutritional factor*	Crude Model	
	OR (95%CI)	p
**Coffee, cups/day**		
None or <1 cup	Reference	
1 cup	0.86 (0.79, 0.93)	<0.001
2-3 cups	0.85 (0.79, 0.91)	<0.001
≥ 4 cups	0.92 (0.90, 0.97)	0.007
**Tea, cups/day**		
None or <1 cup	Reference	
1 cup	0.89 (0.79, 0.099)	0.042
2-3 cups	0.93 (0.85, 1.00)	0.060
≥ 4 cups	1.02 (0.94, 1.10)	0.6871
**Oily fish, servings/day**		
Quartile 1 (0-<0.07)	Reference	
Quartile 2 (0.07- <0.14)	0.87 (0.80, 0.95)	0.002
Quartiles 3 and 4 (≥0.14)	0.86 (0.79, 0.93)	<0.001
**Processed meat, servings/day**		
Quartile 1 (0-<0.07)	Reference	
Quartile 2 (0.07- <0.14)	1.08 (0.96, 1.20)	0.190
Quartile 3 (0.14- <0.43)	1.17 (1.05, 1.31)	0.005
Quartile 4 (≥0.43)	1.25 (1.12, 1.40)	<0.001
**Red meat, servings/day**		
Quartile 1 (0- <0.21)	Reference	
Quartile 2 (0.21- <0.28)	0.96 (0.88, 1.04)	0.280
Quartile 3 (0.28- <0.35)	1.03 (0.94, 1.12)	0.571
Quartile 4 (≥0.35)	1.00 (0.92, 1.08)	0.896
**Fruit (fresh/dried), servings/day**		
Quartile 1 (0- <1.00)	Reference	
Quartile 2 (1.00- <2.25)	1.02 (0.92, 1.12)	0.766
Quartile 3 (2.25- <4.00)	0.92 (0.83, 1.03)	0.155
Quartile 4 (≥4.00)	0.93 (0.84, 1.03)	0.166
**Vegetables (cooked/raw), servings/day**		
Quartile 1 (0- <0.50)	Reference	
Quartile 2 (0.50- <0.67)	0.89 (0.82, 0.96)	0.003
Quartile 3 (0.67- <1.00)	0.85 (0.77, 0.94)	0.001
Quartile 4 (≥1.00)	0.90 (0.83, 0.97)	0.008
**Breastfed as a baby**		
No	Reference	
Yes	0.87 (0.81, 0.93)	<0.001
Don't know	0.99 (0.81, 1.07)	0.735

***** Individual diet factors assessed in separate models.

Supplemental Table 5: Adjusted* OR (95% CI) of having positive COVID-19 test by nutritional factors, stratified by Wave

Nutritional Factor	Wave 1		Wave 2	
	OR (95% CI)	p	OR (95% CI)	p
**Coffee, cups/day**				
None or <1 cup	Reference		Reference	
1 cup	0.89(0.76, 1.04)	0.150	0.92(0.83, 1.01)	0.064
2-3 cups	0.92(0.79, 1.06)	0.247	0.88(0.81, 0.96)	0.005
≥ 4 cups	1.01(0.86, 1.20)	0.888	0.90(0.81, 0.99)	0.035
**Tea, cups/day**				
None or <1 cup	Reference		Reference	
1 cup	0.88(0.70, 1.11)	0.287	0.97(0.85, 1.12)	0.693
2-3 cups	0.95(0.80, 1.12)	0.529	0.92(0.84, 1.02)	0.126
≥ 4 cups	1.03(0.87, 1.21)	0.749	0.96(0.87, 1.06)	0.439
**Oily fish, servings/day**				
Quartile 1 (0-<0.07)	Reference		Reference	
Quartile 2 (0.07- <0.14)	0.91(0.76, 1.09)	0.296	0.94(0.85, 1.05)	0.299
Quartiles 3 and 4 (≥0.14)	0.96(0.81, 1.15)	0.668	0.99(0.89, 1.10)	0.782
**Processed meat, servings/day**				
Quartile 1 (0-<0.07)	Reference		Reference	
Quartile 2 (0.07- <0.14)	0.94(0.75, 1.18)	0.573	1.06(0.92, 1.23)	0.390
Quartile 3 (0.14- <0.43)	1.03(0.81, 1.30)	0.832	1.08(0.94, 1.25)	0.280
Quartile 4 (≥0.43)	1.04(0.83, 1.32)	0.719	1.13(0.98, 1.31)	0.102
**Red meat, servings/day**				
Quartile 1 (0- <0.21)	Reference		Reference	
Quartile 2 (0.21- <0.28)	0.96(0.80, 1.14)	0.636	0.95(0.86, 1.05)	0.335
Quartile 3 (0.28- <0.35)	1.01(0.83, 1.22)	0.935	1.00(0.89, 1.12)	0.938
Quartile 4 (≥0.35)	0.97(0.81, 1.15)	0.721	0.99(0.89, 1.10)	0.839
**Fruit (fresh/dried), servings/day**				
Quartile 1 (0- <1.00)	Reference		Reference	
Quartile 2 (1.00- <2.25)	0.94(0.77, 1.14)	0.522	1.07(0.95, 1.21)	0.270
Quartile 3 (2.25- <4.00)	1.06(0.85, 1.32)	0.607	0.99(0.87, 1.13)	0.884
Quartile 4 (≥4.00)	1.05(0.85, 1.30)	0.672	1.01(0.89, 1.15)	0.871
**Vegetables (cooked/raw), servings/day**				
Quartile 1 (0- <0.50)	Reference		Reference	
Quartile 2 (0.50- <0.67)	0.85(0.72, 1.00)	0.044	0.96(0.87, 1.05)	0.347
Quartile 3 (0.67- <1.00)	0.86(0.70, 1.04)	0.122	0.91(0.81, 1.02)	0.105
Quartile 4 (≥1.00)	0.94(0.79, 1.10)	0.424	0.91(0.83, 1.01)	0.079
**Breastfed as a baby**				
No	Reference		Reference	
Yes	0.91(0.79, 1.05)	0.182	0.93(0.85, 1.01)	0.071
Don’t know	0.93(0.79, 1.10)	0.374	1.02(0.92, 1.12)	0.751

*Adjusted for Townsend deprivation index, baseline age, sex, race, education, income, employment status, home ownership, number of co-habitants, BMI level, smoking status, physical activity, self-rated

health, cholesterol-lowering medication use, antihypertension medication use, history of diabetes, and history of cardiovascular disease, with all diet factors included in the model.

Supplemental Table 6: Adjusted* OR (95% CI) of having positive COVID-19 test by nutritional factors, stratified by baseline age group

Nutritional factor	Baseline Age <55		Baseline Age ≥ 55	
	OR (95%CI)	p	OR (95%CI)	p
**Coffee, cups/day**				
None or <1 cup	Reference		Reference	
1 cup	0.88 (0.78, 0.98)	0.026	0.94 (0.84, 1.06)	0.316
2-3 cups	0.85 (0.77, 0.95)	0.004	0.95 (0.86, 1.06)	0.340
≥ 4 cups	0.88 (0.78, 0.99)	0.040	0.97 (0.85, 1.09)	0.574
**Tea, cups/day**				
None or <1 cup	Reference		Reference	
1 cup	0.87 (0.74, 1.03)	0.107	0.99 (0.83, 1.17)	0.877
2-3 cups	0.96 (0.85, 1.08)	0.515	0.88 (0.78, 0.99)	0.040
≥ 4 cups	1.01 (0.90, 1.14)	0.846	0.94 (0.83, 1.05)	0.279
**Oily fish, servings/day**				
Quartile 1 (0-<0.07)	Reference		Reference	
Quartile 2 (0.07- <0.14)	0.93 (0.82, 1.05)	0.237	0.95 (0.82, 1.09)	0.469
Quartiles 3 and 4 (≥0.14)	0.98 (0.87, 1.11)	0.762	0.99 (0.86, 1.13)	0.837
**Processed meat, servings/day**				
Quartile 1 (0-<0.07)	Reference		Reference	
Quartile 2 (0.07- <0.14)	1.13 (0.95, 1.34)	0.177	1.00 (0.85, 1.19)	0.970
Quartile 3 (0.14- <0.43)	1.13 (0.95, 1.35)	0.172	1.07 (0.90, 1.27)	0.424
Quartile 4 (≥0.43)	1.26 (1.05, 1.50)	0.012	1.06 (0.89, 1.26)	0.513
**Red meat, servings/day**				
Quartile 1 (0- <0.21)	Reference		Reference	
Quartile 2 (0.21- <0.28)	0.99 (0.87, 1.12)	0.855	0.92 (0.81, 1.04)	0.191
Quartile 3 (0.28- <0.35)	1.01 (0.88, 1.16)	0.853	0.99 (0.86, 1.14)	0.860
Quartile 4 (≥0.35)	0.94 (0.83, 1.07)	0.326	1.02 (0.90, 1.16)	0.794
**Fruit (fresh/dried), servings/day**				
Quartile 1 (0- <1.00)	Reference		Reference	
Quartile 2 (1.00- <2.25)	1.04 (0.90, 1.19)	0.609	1.05 (0.90, 1.22)	0.543
Quartile 3 (2.25- <4.00)	0.98 (0.83, 1.15)	0.772	1.06 (0.90, 1.25)	0.503
Quartile 4 (≥4.00)	1.03 (0.88, 1.20)	0.751	1.03 (0.88, 1.21)	0.712
**Vegetables (cooked/raw), servings/day**				
Quartile 1 (0- <0.50)	Reference		Reference	
Quartile 2 (0.50- <0.67)	0.90 (0.81, 1.01)	0.074	0.94 (0.84, 1.06)	0.313
Quartile 3 (0.67- <1.00)	0.88 (0.77, 1.02)	0.096	0.88 (0.76, 1.01)	0.073
Quartile 4 (≥1.00)	0.99 (0.88, 1.12)	0.877	0.86 (0.76, 0.98)	0.020
**Breastfed as a baby**				
No	Reference		Reference	
Yes	0.95 (0.84, 1.08)	0.425	1.02 (0.90, 1.16)	0.717
Don't know	0.91 (0.83, 1.00)	0.052	0.94 (0.84, 1.05)	0.294

* Adjusted for Townsend deprivation index, baseline age, sex, race, education, income, employment status, home ownership, number of co-habitants, BMI level, smoking status, physical activity, self-rated health, cholesterol-lowering medication use, antihypertension medication use, history of diabetes, and history of cardiovascular disease, with all diet factors included in the model (i.e. mutual adjustment).

Supplemental Table 7: Adjusted* OR (95% CI) of having positive COVID-19 test by nutritional factors, stratified by sex

Nutritional factor	Female		Male	
	OR (95%CI)	p	OR (95%CI)	p
**Coffee, cups/day**				
None or <1 cup	Reference		Reference	
1 cup	0.90 (0.81, 1.01)	0.073	0.90 (0.80, 1.01)	0.080
2-3 cups	0.91 (0.82, 1.00)	0.060	0.88 (0.79, 0.98)	0.016
≥ 4 cups	0.92 (0.81, 1.03)	0.155	0.91 (0.81, 1.03)	0.136
**Tea, cups/day**				
None or <1 cup	Reference		Reference	
1 cup	0.90 (0.76, 1.06)	0.207	0.96 (0.81, 1.13)	0.598
2-3 cups	0.90 (0.80, 1.02)	0.096	0.95 (0.84, 1.08)	0.459
≥ 4 cups	0.96 (0.85, 1.07)	0.444	1.00 (0.88, 1.12)	0.945
**Oily fish, servings/day**				
Quartile 1 (0-<0.07)	Reference		Reference	
Quartile 2 (0.07- <0.14)	0.99 (0.86, 1.13)	0.824	0.90 (0.79, 1.03)	0.131
Quartiles 3 and 4 (≥0.14)	1.04 (0.91, 1.18)	0.603	0.94 (0.82, 1.06)	0.313
**Processed meat, servings/day**				
Quartile 1 (0-<0.07)	Reference		Reference	
Quartile 2 (0.07- <0.14)	1.06 (0.92, 1.23)	0.415	1.01 (0.81, 1.24)	0.965
Quartile 3 (0.14- <0.43)	1.09 (0.94, 1.28)	0.250	1.07 (0.87, 1.32)	0.538
Quartile 4 (≥0.43)	1.16 (0.99, 1.36)	0.066	1.11 (0.90, 1.36)	0.343
**Red meat, servings/day**				
Quartile 1 (0- <0.21)	Reference		Reference	
Quartile 2 (0.21- <0.28)	1.04 (0.92, 1.17)	0.542	0.83 (0.73, 0.95)	0.008
Quartile 3 (0.28- <0.35)	1.07 (0.93, 1.22)	0.353	0.89 (0.77, 1.03)	0.126
Quartile 4 (≥0.35)	1.08 (0.96, 1.22)	0.196	0.84 (0.74, 0.97)	0.013
**Fruit (fresh/dried), servings/day**				
Quartile 1 (0- <1.00)	Reference		Reference	
Quartile 2 (1.00- <2.25)	0.95 (0.81, 1.11)	0.506	1.12 (0.98, 1.28)	0.092
Quartile 3 (2.25- <4.00)	0.88 (0.74, 1.05)	0.160	1.14 (0.98, 1.33)	0.097
Quartile 4 (≥4.00)	0.88 (0.75, 1.05)	0.148	1.15 (0.99, 1.34)	0.072
**Vegetables (cooked/raw), servings/day**				
Quartile 1 (0- <0.50)	Reference		Reference	
Quartile 2 (0.50- <0.67)	0.89 (0.79, 1.01)	0.061	0.97 (0.87, 1.08)	0.524
Quartile 3 (0.67- <1.00)	0.87 (0.76, 1.01)	0.067	0.89 (0.78, 1.03)	0.119
Quartile 4 (≥1.00)	0.96 (0.84, 1.08)	0.486	0.87 (0.77, 0.98)	0.018
**Breastfed as a baby**				
No	Reference		Reference	
Yes	0.95 (0.85, 1.07)	0.425	1.02 (0.90, 1.15)	0.737
Don't know	0.89 (0.82, 0.98)	0.018	0.94 (0.84, 1.05)	0.281

***** Adjusted for Townsend deprivation index, baseline age, race, education, income, employment status, home ownership, number of co-habitants, BMI level, smoking status, physical activity, self-rated health, cholesterol-lowering medication use, antihypertension medication use, history of diabetes, and history of cardiovascular disease, with all diet factors included in the model (i.e. mutual adjustment).

Supplemental Table 8: Adjusted* OR (95% CI) of having positive COVID-19 test by nutritional factors, stratified by racial group

Nutritional factor**	White		Asian		Black		Mixed/Others	
	OR (95%CI)	p	OR (95%CI)	p	OR (95%CI)	p	OR (95%CI)	p
**Coffee, cups/day**								
None or <1cup	Reference		Reference		Reference		Reference	
1 cup	0.91 (0.84, 0.99)	0.034	1.04 (0.68, 1.59)	0.865	0.45 (0.25, 0.82)	0.009	0.88 (0.49, 1.59)	0.669
2-3 cups	0.91 (0.84, 0.98)	0.017	0.85 (0.50, 1.43)	0.535	0.44 (0.22, 0.90)	0.024	0.56 (0.30, 1.03)	0.061
≥ 4 cups	0.92 (0.84, 1.00)	0.060	1.88 (0.81, 4.37)	0.141	0.59 (0.24, 1.49)	0.268	0.58 (0.27, 1.22)	0.149
**Tea, cups/day**								
None or <1cup	Reference		Reference		Reference		Reference	
1 cup	0.91 (0.80, 1.03)	0.126	2.21 (0.99, 4.94)	0.054	1.19 (0.52, 2.70)	0.686	0.88 (0.38, 2.03)	0.771
2-3 cups	0.91 (0.83, 1.00)	0.042	1.93 (0.94, 3.97)	0.073	1.05 (0.53, 2.07)	0.886	0.73 (0.36, 1.46)	0.372
≥ 4 cups	0.95 (0.88, 1.04)	0.272	1.90 (0.91, 3.96)	0.087	1.39 (0.69, 2.81)	0.361	1.21 (0.62, 2.35)	0.573
**Oily fish, servings/day**								
Quartile 1	Reference		Reference		Reference		Reference	
Quartile 2	0.93 (0.84, 1.02)	0.130	0.97 (0.56, 1.69)	0.922	0.86 (0.34, 2.17)	0.744	1.33 (0.56, 3.16)	0.522
Quartiles 3 and 4	0.97 (0.88, 1.06)	0.506	1.13 (0.67, 1.90)	0.657	0.91 (0.37, 2.21)	0.831	1.34 (0.58, 3.07)	0.491
**Processed meat, servings/day**								
Quartile 1	Reference		Reference		Reference		Reference	
Quartile 2	1.08 (0.95, 1.23)	0.237	0.76 (0.43, 1.34)	0.338	0.43 (0.20, 0.95)	0.036	1.14 (0.54, 2.43)	0.726
Quartile 3	1.13 (0.99, 1.29)	0.081	0.82 (0.45, 1.49)	0.506	0.47 (0.21, 1.07)	0.072	0.88 (0.38, 2.06)	0.768
Quartile 4	1.16 (1.02, 1.33)	0.028	0.70 (0.38, 1.29)	0.251	1.01 (0.43, 2.37)	0.975	1.23 (0.54, 2.80)	0.631
**Red meat, servings/day**								
Quartile 1	Reference		Reference		Reference		Reference	
Quartile 2	0.95 (0.87, 1.04)	0.282	1.38 (0.64, 2.96)	0.412	0.97 (0.41, 2.29)	0.938	1.62 (0.76, 3.43)	0.209
Quartile 3	0.98 (0.88, 1.08)	0.682	1.45 (0.74, 2.83)	0.281	1.48 (0.58, 3.76)	0.413	1.75 (0.80, 3.81)	0.160
Quartile 4	0.98 (0.89, 1.07)	0.625	1.27 (0.63, 2.54)	0.506	1.36 (0.53, 3.47)	0.518	1.62 (0.79, 3.32)	0.192
**Fruit (fresh/dried), servings/day**								
Quartile 1	Reference		Reference		Reference		Reference	
Quartile 2	1.04 (0.93, 1.15)	0.498	1.68 (0.76, 3.73)	0.199	1.17 (0.44, 3.13)	0.751	0.61 (0.26, 1.42)	0.253
Quartile 3	1.02 (0.90, 1.15)	0.770	1.26 (0.53, 2.99)	0.595	1.36 (0.47, 3.92)	0.569	0.52 (0.21, 1.32)	0.170

Quartile 4	1.01 (0.90, 1.13)	0.899	1.97 (0.88, 4.43)	0.101	1.00 (0.36, 2.77)	0.996	0.65 (0.27, 1.55)	0.330
**Vegetables (cooked/raw), servings/day**								
Quartile 1	Reference		Reference		Reference		Reference	
Quartile 2	0.90 (0.83, 0.98)	0.011	2.04 (1.15, 3.61)	0.015	1.25 (0.65, 2.43)	0.502	1.40 (0.66, 2.94)	0.378
Quartile 3	0.85 (0.77, 0.94)	0.002	1.76 (0.99, 3.13)	0.055	1.55 (0.78, 3.08)	0.214	1.85 (0.91, 3.75)	0.087
Quartile 4	0.89 (0.82, 0.97)	0.011	1.71 (0.95, 3.09)	0.075	1.10 (0.51, 2.35)	0.816	1.28 (0.57, 2.87)	0.552
**Breastfed as a baby**								
No	Reference		Reference		Reference		Reference	
Yes	0.98 (0.90, 1.07)	0.618	1.71 (0.69, 4.24)	0.247	1.02 (0.34, 3.10)	0.975	1.17 (0.54, 2.51)	0.690
Don’t know	0.91 (0.85, 0.98)	0.010	2.14 (0.96, 4.78)	0.064	0.45 (0.17, 1.21)	0.112	0.78 (0.41, 1.52)	0.468

***** Adjusted for Townsend deprivation index, baseline age, race, education, income, employment status, home ownership, number of co-habitants, BMI level, smoking status, physical activity, self-rated health, cholesterol-lowering medication use, antihypertension medication use, history of diabetes, and history of cardiovascular disease, with all diet factors included in the model (i.e. mutual adjustment).

**Race-specific cut-points were used to determine quartiles.

Supplemental Figure 1. Flow chart depicting the study design and the analysis sample

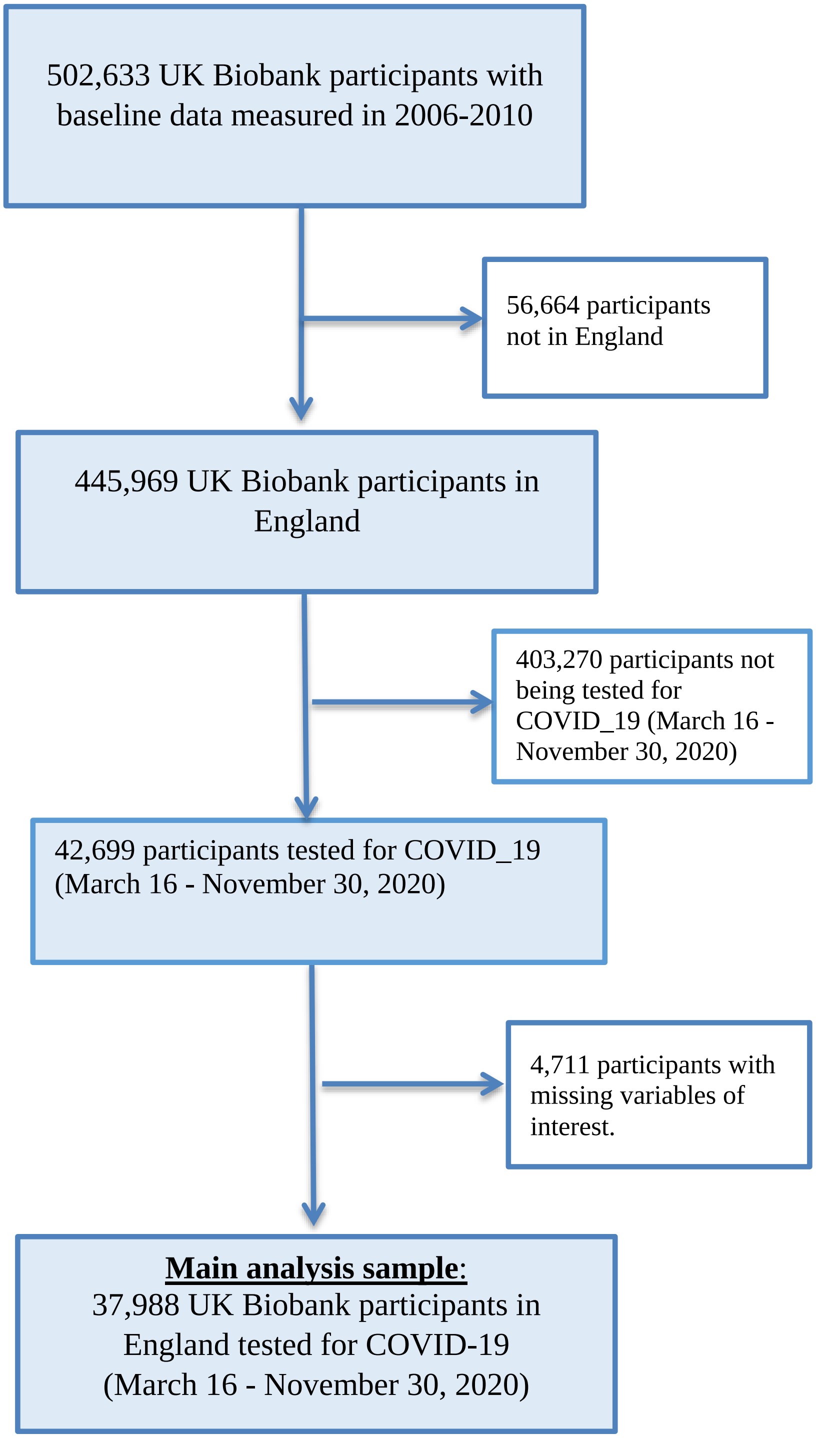

Supplemental Figure 2: National (UK) COVID-19 Cases Reported Between March and December 1 2020

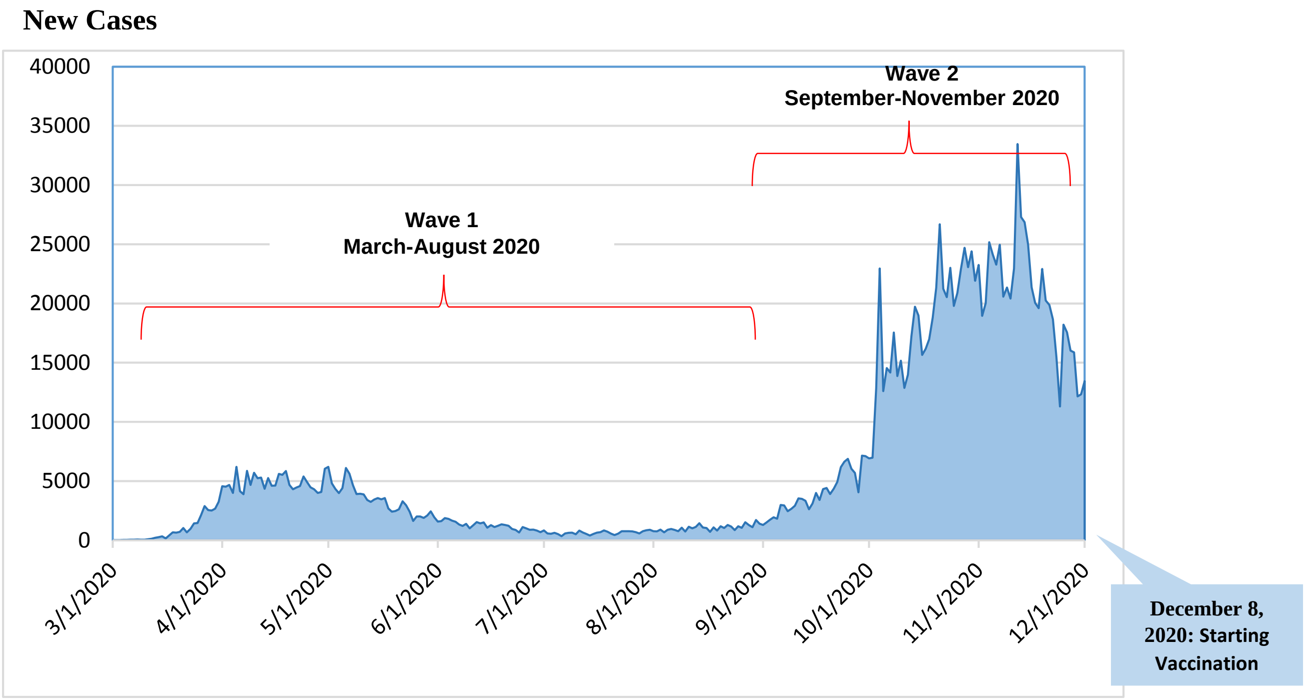

Source: Cases by date reported https://coronavirus.data.gov.uk/details/cases. Accessed June 1, 2021

Supplemental Figure 3. Total COVID-19 Positive Tests in UKB (A) Compared to National Data by Wave (B and C)*

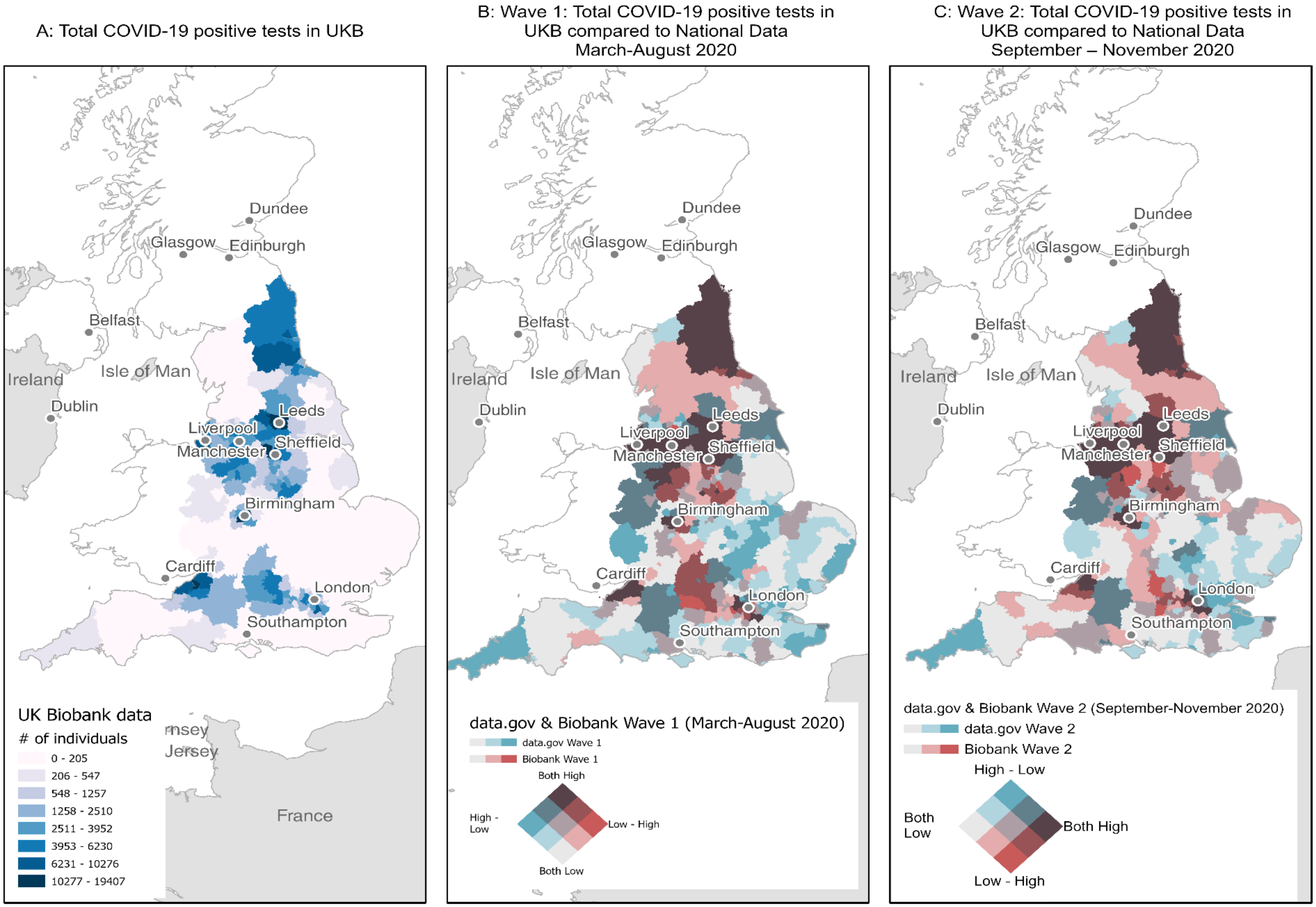

* The UKB geo-data and National (UK) COVID-19 surveillance data was imported, projected, and converted to be compatible with each other in ArcGIS for visual inspection (see COVID-19 exposure in Materials and Methods section for more details). **Wave 1**: Total positive tests for March-August 2020; **Wave 2:** Total positive tests for September – November 2020; Source: UKB and https://coronavirus.data.gov.uk/ accessed April 4, 2021.
